# Development and In Vitro Evaluation of [^64^Cu]Cu-NOTA-TP-PSMA, a Novel Radiotheranostic Agent Against Prostate Cancer

**DOI:** 10.3390/ijms262311651

**Published:** 2025-12-01

**Authors:** Hoda Talebian, Samia Ait-Mohand, Prenitha Mercy Ignatius Arokia Doss, Léon Sanche, Brigitte Guérin

**Affiliations:** 1 Department of Medical Imaging and Radiation Sciences, Faculty of Medicine and Health Sciences, Université de Sherbrooke, Sherbrooke, QC J1H 5N4, Canadaleon.sanche@usherbrooke.ca (L.S.); 2 Institut de Recherche sur le Cancer de l’Université de Sherbrooke (IRCUS), Université de Sherbrooke, Sherbrooke, QC J1H 5N4, Canada; 3 Sherbrooke Molecular Imaging Center (CIMS), Centre de Recherche du CHUS (CRCHUS), Sherbrooke, QC J1H 5H3, Canada

**Keywords:** prostate cancer, PSMA, radiotheranostic agent, copper-64, terpyridine-platinum, in vitro validation

## Abstract

Prostate cancer (PCa), particularly in its metastatic form, remains a major clinical challenge due to limited diagnostic and therapeutic options. To address this, we developed a novel radiotheranostic agent, [^64^Cu]Cu-NOTA-TP-PSMA, by conjugating a prostate-specific membrane antigen (PSMA) ligand to a ^64^Cu-radiolabeled terpyridine-platinum (TP) compound previously shown to exert selective cytotoxicity against cancer cells. In this study, the biological performance of [^64^Cu]Cu-NOTA-TP-PSMA was compared with the monomeric analogs [^64^Cu]Cu-NOTA-PSMA and [^64^Cu]Cu-NOTA-TP through in vitro studies in PSMA-positive LNCaP prostate cancer cells and non-malignant HEK-293 cells. [^64^Cu]Cu-NOTA-TP-PSMA showed high stability, PSMA binding affinity and exhibited substantially enhanced uptake, internalization, retention, and nuclear localization in LNCaP cells relative to the monomers, whereas uptake and nuclear accumulation in HEK-293 cells were negligible. Cytotoxicity assays further demonstrated potent and selective activity in LNCaP cells, with EC_50_ values in the low nanomolar range, and minimal toxicity in HEK-293 cells. Collectively, these results identify [^64^Cu]Cu-NOTA-TP-PSMA as a promising radiotheranostic agent, warranting further in vivo evaluation for prostate cancer imaging and targeted radiotherapy.

## 1. Introduction

Prostate cancer (PCa) is the most commonly diagnosed non-cutaneous adenocarcinoma and ranks the second most frequently diagnosed cancer in men and ranks fifth among causes of cancer-related mortality worldwide [1]. Early-stage localized PCa generally responds well to radical prostatectomy [2]. However, delayed diagnosis often leads to metastasis in the bones or lymph nodes, reducing treatment efficacy [3]. Incomplete tumor removal at early detection may also elevate the risk of recurrence [4]. As the disease progresses, androgen deprivation therapy is commonly used for initial control. Nevertheless, this often leads to the development of metastatic castration-resistant prostate cancer (mCRPC), wherein the 5-year survival rate declines sharply to approximately 30% [5]. This underscores the critical importance of early detection and timely intervention. 

Although PCa diagnostics have evolved, traditional methods such as digital rectal examination, prostate-specific antigen (PSA) testing, and biopsy still suffer from limitations, including patient discomfort, low specificity, and infection risk [6,7]. This underscores the need for advanced diagnostic strategies that allow for early detection. Treatment modalities for PCa range from monotherapy to multimodal strategies, often guided by tumor stage, Gleason score, and PSA levels [8]. Targeted theranostic approaches, particularly those using positron emission tomography (PET), imaging tracers and radiotherapeutic agents, have emerged as promising approaches. These latter employ radiolabeled small molecules that bind selectively to PCa lesions, enabling both imaging and therapy [9,10]. 

The prostate-specific membrane antigen (PSMA) is a membrane-bound enzyme, markedly overexpressed in PCa [11]. To exploit this biomarker that emerged as a key target for both imaging and radioligand therapy, researchers have developed small-molecule PSMA inhibitors, most notably ureido-linked dipeptides [12,13,14]. Among these, gallium-68 [^68^Ga]Ga-PSMA-11 has gained prominence and is now regarded as the diagnostic gold standard [15]. In therapy, agents such as lutetium-177 [^177^Lu]Lu-PSMA-617 and [^177^Lu]Lu-PSMA I&T leverage the 6.65-day half-life and β-emission of ^177^Lu to deliver targeted cytotoxicity while minimizing damage to healthy tissues [16]. Despite [^177^Lu]Lu-PSMA-617 receiving FDA approval in 2022, its use has been limited by production challenges, with only modest survival benefits, and the emergence of resistance [17]. Alpha-emitting actinium-225 [^225^Ac]Ac-PSMA-617 shows promise but suffers from toxicity (e.g., xerostomia) and limited availability [18]. Moreover, [^177^Lu]Lu-PSMA can be used for SPECT imaging, it typically requires relatively high activity for detectable signal because only a small fraction of its decay energy is suitable for imaging. Therefore, its usefulness for SPECT is limited and often less sensitive than other radionuclides. [^225^Ac]Ac-PSMA-617 lacks PET compatibility, necessitating separate diagnostic agents (e.g., [^68^Ga]Ga-PSMA-11) [19]. Despite these drawbacks of current PSMA-targeted theranostics, advancing radioligand therapy (RLT) and imaging strategies remains essential.

Traditional platinum-based chemotherapies exert their anticancer effects by disrupting DNA through G-quadruplex (G4) binding; however, these treatments are limited by systemic toxicity and the emergence of multidrug resistance [20]. To overcome some of these issues, terpyridine–platinum (TP) complexes have been developed, which address these limitations to an extent but still face challenges related to therapeutic efficacy [21,22]. On the other hand, copper-64 (^64^Cu) is widely recognized as a radionuclide with notable advantages, including ease of production via cyclotron access and a unique dual decay mode involving both β^+^ emission, which is suitable for imaging, and therapeutic emissions of β^−^ particles and Auger electrons. This combination makes ^64^Cu highly effective for both diagnostic imaging and targeted radiotherapy [23,24]. Building on this, we previously developed the [^64^Cu]Cu-NOTA-TP radiotheranostic. agent, which demonstrated a remarkable 55,000-fold increase in cytotoxicity against cancer cells compared to conventional platinum therapies, along with confirmed stability, telomeric G4-mediated DNA intercalation, and effective tumor uptake [25,26].

Through the emission of Auger electrons and positrons, ^64^Cu acts as a highly localized source of low-energy electrons (LEEs), which are particularly effective at inducing clustered DNA damage in sensitive genomic regions, ultimately triggering cell death [25]. Our previous studies indicate that the DNA lesions generated by these LEEs are maximized when combined with the structural perturbations caused by the platinum-based TP intercalating into G4 DNA [25]. This dual mechanism—radiation-induced DNA strand breaks synergizing with chemically induced DNA destabilization—likely underlies the observed supra-additive and selective cytotoxicity. However, the current design may not fully exploit this synergy. By coupling [^64^Cu]Cu-NOTA-TP to a PSMA-targeting moiety, we expect enhanced specificity for PCa cells and amplified chemical and radiobiological effects, thereby improving therapeutic efficacy. 

**Figure 1:**
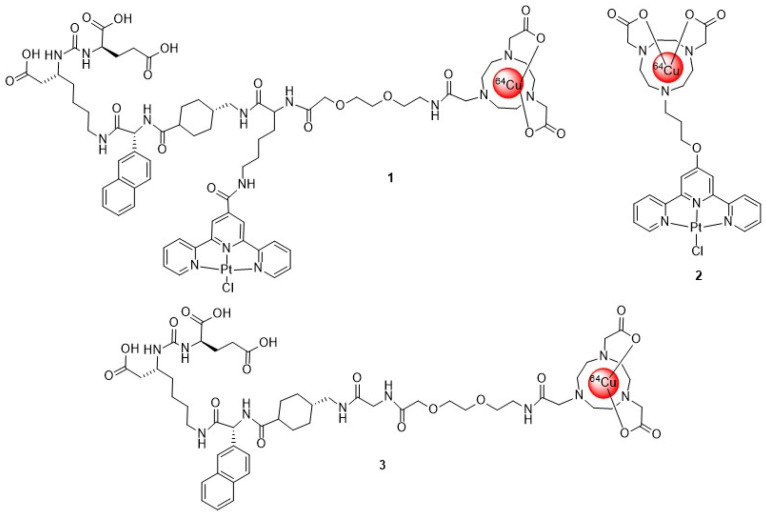
The chemical structures of [^64^Cu]Cu-NOTA-TP-PSMA **1**, [^64^Cu]Cu-NOTA-TP **2** and [^64^Cu]Cu-NOTA-PSMA **3**.

In this study, we report the preparation of the novel radiotheranostic agent [^64^Cu]Cu-NOTA-TP-PSMA **1** and evaluate its in vitro selectivity, internalization, and cytotoxicity on PSMA-positive and normal cells. Comparisons with the monomeric analogs [^64^Cu]Cu-NOTA-TP **2** and [^64^Cu]Cu-NOTA-PSMA **3** (Figure 1) provide further insight into the specificity and cytotoxicity of this compound.

## 2. Results

### 2.1. Synthesis, Radiolabling and Characterization of ^64^Cu-NOTA-TP-PSMA

**Scheme 1:**
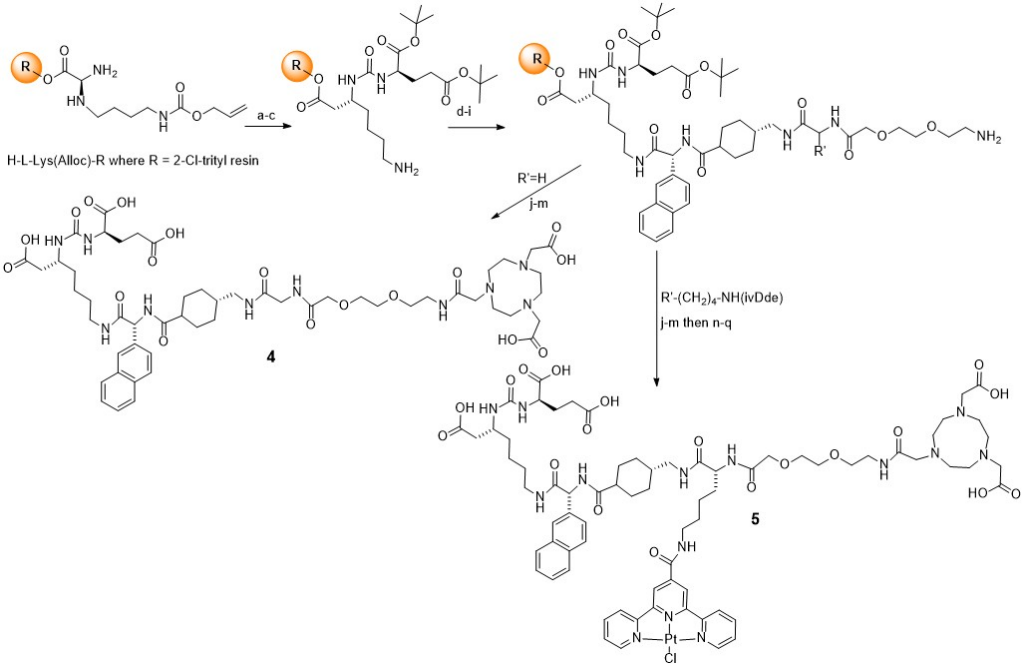
Synthesis of NOTA-PSMA **4** and NOTA-TP-PSMA **5**. ***Reagents and conditions:*** (a) DSC, DIPEA, DM; (b) H-Glu(OtBu)_2_ HCl; (c) Pd(PPh_3_)_4_, morpholine, DCM; (d) Fmoc-AA-OH, HATU, DIEA, DMF; (e) Piperidine, DMF; (f) Fmoc-Gly-OH, HATU, DIPEA, (R’=H); or Fmoc-Lys(ivDde)-OH, HATU, DIPEA (R’=(CH_2_)_4_-NH(ivDde); (g) Piperidine, DMF; (h) Fmoc-PEG-OH, HATU, DIPEA; (i) Piperidine, DMF; (j) bromoacetic acid, EDC, DCM; (k) TACN, DCM; (l) *tert*butyl bromoacetate, DIPEA, *N*-methylpyrrolidone; (m) TFA, H_2_O, TIPS; (n) 2% NH_2_NH_2_, DMF; (o) 2,2′:6′,2′′-terpyridine-4′-carboxylic acid, HATU, DIPEA, DMF; (p) TFE/DCM (1/2), (q) K_2_PtCl_4_, MeOH; TFA (neat). R = 2-chlorotrityl-chloride resin.

NOTA-PSMA **4** and NOTA-TP-PSMA **5** peptides were successfully synthesized using standard Fmoc-based solid-phase peptide synthesis (SPPS). The synthetic route yielded the desired products in 38% and 36% overall yield, respectively (Scheme 1, Figures S1 and S2). Coordination of natural copper (^nat^Cu) to both ligands proceeded efficiently, with excellent complexation yields confirmed by analytical characterization (Figures S3 and S4). While ^nat^Cu-NOTA-PSMA was readily soluble in aqueous media, ^nat^Cu-NOTA-TP-PSMA required formulation in 5% DMSO in saline due to reduced solubility attributed to the increased hydrophobicity conferred by the TP moiety.

Radiolabeling with [^64^Cu]Cu(OAc)_2_ was rapid and highly efficient, affording radiochemical conversions exceeding 99% without the need for post-labeling purification (Figure S5A,B). The labeled products were obtained with high apparent molar activities, measured at 115–225 MBq/nmol for [^64^Cu]Cu-NOTA-TP-PSMA **1** and 100–115 MBq/nmol for [^64^Cu]Cu-NOTA-PSMA **2**, highlighting their suitability for in vitro and further in vivo applications.

### 2.2. Stability of ^64^Cu-NOTA-TP-PSMA

Incubation of [^64^Cu]Cu–NOTA–TP–PSMA 1 in mouse plasma at 37 °C for 24 h revealed strong plasma protein association, with approximately 91% of the total radioactivity remaining bound to plasma proteins. Analysis of the protein-free supernatant by radio-iTLC (Figure S5C) showed no evidence of free ^64^Cu^2+^, indicating minimal degradation of the radiocomplex under physiological conditions. 

### 2.3. Competitive Binding Assay

The competitive binding assay was performed in LNCaP cells to evaluate the PSMA-binding affinity of the non-radioactive analogs, ^nat^Cu-NOTA-PSMA and ^nat^Cu-NOTA-TP-PSMA using [^64^Cu]Cu-NOTA-PSMA as the radioligand. The resulting IC_50_ values were 40.8 ± 14.9 nM for ^nat^Cu-NOTA-PSMA and 30.6 ± 16.6 nM for ^nat^Cu-NOTA-TP-PSMA (Table 1, entries 1–2, Figure S6). The addition of the TP moiety did not significantly impair PSMA affinity. 

### 2.4. Kinetics of Cellular Uptake, Internalization, and Efflux

**Figure 2:**
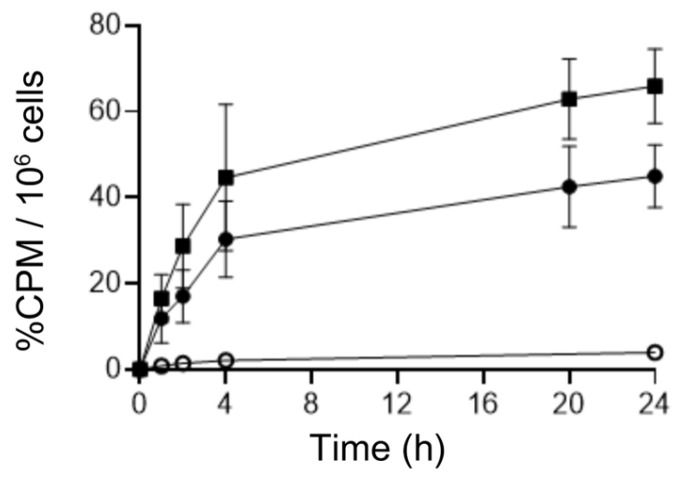
Uptake at 37 °C (black square), internalization at 37 °C (black circle) and at 4 °C (white circle) kinetics caried out with [^64^Cu]Cu-NOTA-TP-PSMA **1** on LNCaP cells over 24 h. Data are presented as the percentage of total activity (CPM) per 10^6^ cells (%CPM/10^6^ cells).

Time-course analysis at 37 °C revealed a progressive increase in total uptake and internalization of [^64^Cu]Cu-NOTA-TP-PSMA **1** over 24 h incubation in LNCaP cells, Figure 2. Uptake increased from 0 to 65.9± 8.6%CPM/10^6^ cells, while internalization rose from 0 to 44.9 ± 7.3%CPM/10^6^ cells. Internalization of [^64^Cu]Cu-NOTA-TP-PSMA in LNCaP cells was significantly reduced at 4 °C compared to 37 °C, with more than a 10-fold decrease observed (3.9 ± 0.5 vs. 44.9 ± 7.3%CPM/10^6^ cells, *p* < 0.05), indicating that internalization is predominantly receptor-dependent (Figure 2). This difference was statistically significant (*p* = 0.02; unpaired *t*-test).

**Figure 3:**
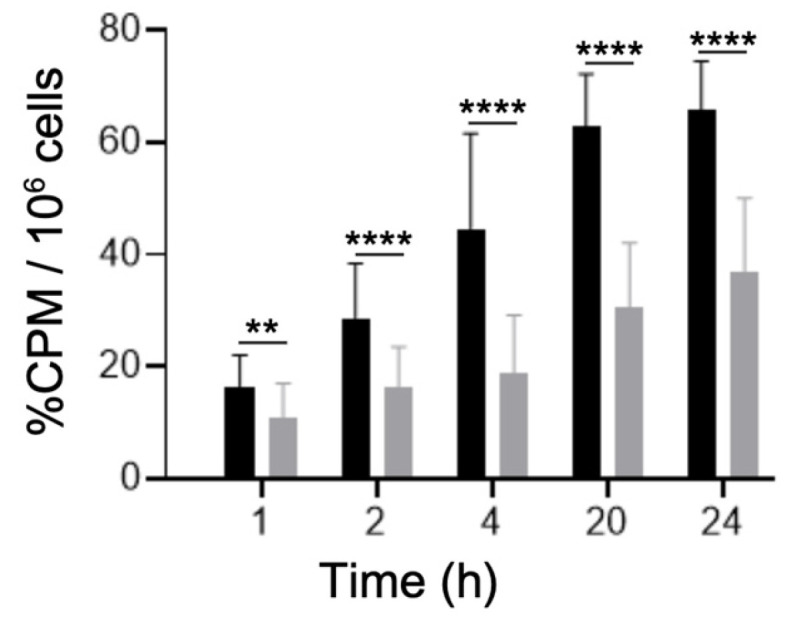
Time-dependent [^64^Cu]Cu-NOTA-TP-PSMA cellular uptake (black bars) vs. inhibited uptake (grey bars) by blocking agent. Data are presented as the %CPM/10^6^ cells. **: *p* < 0.01, ****: *p* < 0.0001.

Blocking studies using a 1000-fold excess showed reduced uptake of [^64^Cu]Cu-NOTA-TP-PSMA **1** (Figure 3, Supplementary Table S1). Statistically significant differences between the uptake under normal and blocking conditions were observed at all time points from 1 to 24 h post incubation (*p* < 0.01, unpaired *t*-tests), confirming PSMA-specific uptake of **1**. 

Selectivity of [^64^Cu]Cu-NOTA-TP-PSMA **1** was further evaluated in non-malignant HEK-293 cells, which exhibited consistently low uptake and internalization over the 24-h period, reaching a maximum of 8.30 ± 1.63%CPM/10^6^ cells for uptake and 7.6 ± 1.7% CPM/10^6^ cells for internalization at 24 h (Supplementary Table S2). In contrast, PSMA-positive LNCaP cells showed significantly higher uptake and internalization, with uptake approximately 8-fold and internalization of approximately 6-fold greater than HEK-293 cells at 24 h (Supplementary Table S2). 

To compare ligand performance, internalization and uptake were assessed for ^64^Cu-labeled conjugates **1**, **2**, and **3**. At 24 h, [^64^Cu]Cu-NOTA-TP-PSMA exhibited the highest uptake and internalization, outperforming both monomeric analogs [^64^Cu]Cu-NOTA-TP **2** and [^64^Cu]Cu-NOTA-PSMA **3** (Table 2, entries 1–3). Statistical analysis (unpaired *t*-test) confirmed that the uptake of **1** was significantly higher than **2** (*p* < 0.01) and **3** (*p* < 0.0001) at 24 h post incubation.

**Figure 4:**
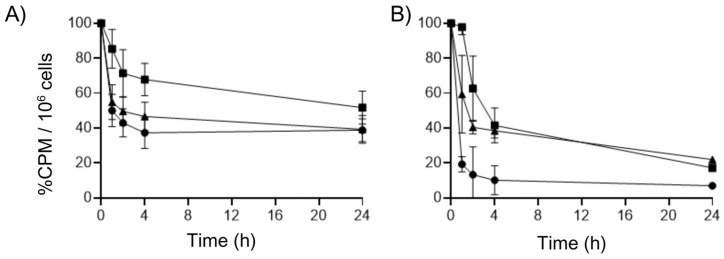
Comparative efflux profiles of ^64^Cu-labeled conjugates over 24 h in LNCaP (**A**) and HEK-293 (**B**). [^64^Cu]Cu-NOTA-TP-PSMA **1** (black square), [^64^Cu]Cu-NOTA-TP **2** (black circle) and [^64^Cu]Cu-NOTA-PSMA **3** (black triangle). Data are presented as the %CPM/10^6^ cells.

Comparative efflux studies were conducted on the ^64^Cu-labeled conjugates in LNCaP cells, Figure 4A. [^64^Cu]Cu-NOTA-TP-PSMA **1** demonstrated higher retention at all time point compared to both [^64^Cu]Cu-NOTA-TP **2** and [^64^Cu]Cu-NOTA-PSMA **3**. In contrast, retention in HEK cells was minimal for all compounds, with **2** showing the lowest retention (Figure 4B, Table S3).

### 2.5. Nuclear Localization

**Figure 5:**
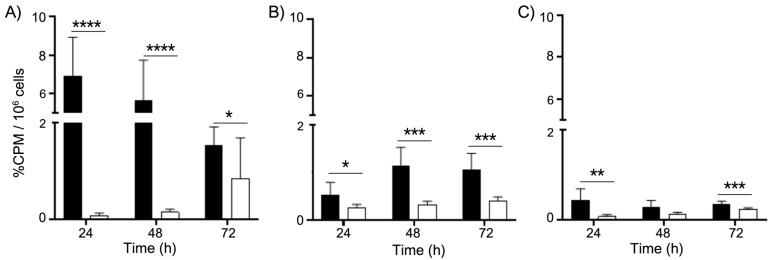
Nuclear uptake of [^64^Cu]Cu-NOTA-TP-PSMA **1** (**A**), [^64^Cu]Cu-NOTA-TP **2** (**B**) and [^64^Cu]Cu-NOTA-PSMA **3** (**C**) in LNCaP (solid black) and HEK-293 (white) at 24-, 48-, and 72-h post incubation. Data are expressed as %CPM/10^6^ cells. *: *p* < 0.05, **: *p* < 0.01, ***: *p* < 0.001, ****: *p* < 0.0001.

At 24 h post incubation, [^64^Cu]Cu-NOTA-TP-PSMA **1** exhibited higher nuclear uptake in LNCaP cells than in HEK-293 cells, representing an 84-fold difference (Figure 5A, Table S4). Uptake in LNCaP cells remained elevated at 48 h, whereas HEK-293 cells showed a 36-fold lower uptake. By 72 h, nuclear uptake decreased in both cell lines, though LNCaP cells maintained slightly higher uptake than HEK-293 cells (1.8-fold). For [^64^Cu]Cu-NOTA-TP **2**, nuclear uptake in LNCaP cells was 0.5 ± 0.2% CPM/10^6^ cells, 7.2-fold higher than that observed in HEK-293 cells (Figure 5B, Table S4). At 48 h, uptake in LNCaP cells increased to 1.2 ± 0.4%, while HEK-293 cells showed 0.3 ± 0.1. At 72 h, uptake in LNCaP remained stable at 1.0 ± 0.3%, compared to 0.4 ± 0.1% in HEK-293 cells, maintaining a 2.6-fold selectivity. In comparison, [^64^Cu]Cu-NOTA-PSMA **3** exhibited low nuclear uptake in LNCaP cells at 24 h (0.4 ± 0.3%), a 5.8-fold increase over HEK-293 cells (Figure 5C, Table S4). At 48 h, uptake was 0.30 ± 0.13% in LNCaP and 0.13 ± 0.05% in HEK-293 cells. By 72 h, nuclear uptake in LNCaP was 0.35 ± 0.07%, while HEK-293 showed 0.23 ± 0.04% (1.5-fold). 

While nuclear uptake in HEK-293 cells remained low for all compounds, [^64^Cu]Cu-NOTA-TP-PSMA **1** consistently demonstrated the highest nuclear uptake in LNCaP ells at all evaluated time points. 

### 2.6. Cytotoxicity

The cytotoxicity of compounds **1**–**3**, ^nat^Cu-NOTA-TP-PSMA, and cisplatin (platinum-based drug) were assessed with PrestoBlue, a resazurin-based method to assess mitochondrial function in LNCaP and HEK-293 cells at 24, 48, and 72 h. Effective concentration (EC_50_) values summarized in Table 3 were calculated from the dose–response curves of each compound (Figure S7).

In LNCaP cells, [^64^Cu]Cu-NOTA-TP-PSMA **1** exhibited greater cytotoxicity, yielding the lowest EC_50_ values across all time points (Table 3, entry 1 vs. entries 2-5). In HEK-293 cells, [^64^Cu]Cu-NOTA-TP-PSMA **1** demonstrated low cytotoxicity, with EC_50_ values consistently exceeding 250 nM. Monomeric compounds **2** and **3** displayed selectivity toward cancer cells but were 5–16 fold less cytotoxic than compound **1** at 24 h post incubation (Table 3, entries 2–3 vs. entry 1). 

Both the non-radioactive analogue ^Nat^Cu-NOTA-TP-PSMA and cisplatin, exhibited minimal cytotoxicity in both cell lines, with EC_50_ values in the micromolar range across all time points (Table 3, entries 4–5).

## 3. Discussion

[^64^Cu]Cu-NOTA-TP-PSMA **1** was developed to overcome limitations in PSMA-targeted radioligand therapy (RLT), such as heterogeneous PSMA expression and resistance to monotherapy [27,28]. To improve therapeutic efficacy and targeting precision, recent efforts have focused on dual-action radiotheranostic agents that integrate selective tumor binding with intracellular and subcellular delivery mechanisms [29]. Building on our prior studies demonstrating the cytotoxic potential of [^64^Cu]Cu-NOTA-TP in colorectal cancer models [25,26], we designed a novel radiotheranostic agent linking a PSMA-targeting ligand to a ^64^Cu-radiolabeled TP moiety, a DNA-affinic metal-binding group.

This study demonstrates that NOTA-PSMA **4** and NOTA-TP-PSMA ***5*** were obtained with a purity exceeding 95% not optimized overall yields of 36–38%. Radiolabeling with [^64^Cu]Cu(OAc)_2_ was rapid and quantitative (>99% conversion), yielding products with high molar activities (100–225 MBq/nmol) suitable for in vitro and in vivo studies. 

Both ^nat^Cu-NOTA-TP-PSMA and ^nat^Cu-NOTA-PSMA exhibited similar nanomolar-range IC_50_ values, indicating comparable binding affinity to PSMA-expressing cells (Table 1). Although these values are slightly higher than those reported for PSMA-617, they remain well within a therapeutically relevant range, supporting their potential clinical utility [29]. Incorporation of TP into the linker between the NOTA chelator and the glutamate-urea-lysine pharmacophore did not compromise PSMA affinity, consistent with previous work showing that strategic modifications at this position can preserve receptor binding [30,31]. Given its aromatic and lipophilic nature, TP could interact the S1-hydrophobic pocket of PSMA [14,32]. 

Temperature-dependent, with ~10-fold lower uptake at 4 °C versus 37 °C and blocking studies supported that [^64^Cu]Cu-NOTA-TP-PSMA **1** internalization is PSMA receptor-specific (Figure 2 and Figure 3) [33]. The higher uptake and internalization of compound **1** in LNCaP cells compared to non-malignant HEK-293 cells across all time points (Table S2) underscores its strong PSMA-targeting specificity and preferential accumulation in prostate cancer cells. 

Our data further show that [^64^Cu]Cu-NOTA-TP-PSMA **1** outperforms its monomeric analogues, with 2.7-fold higher internalization compared to [^64^Cu]Cu-NOTA-PSMA and 1.2-fold higher than [^64^Cu]Cu-NOTA-TP in cancer cells at 24 h (Table 2). This is likely due to dual internalization via both PSMA and TP-mediated pathways. Metal-TP complexes can bind to cell membrane receptors or interact electrostatically with the membrane, triggering endocytosis via clathrin- or caveolae-mediated pathways [34]; after internalization, these complexes are trafficked through endosomes and lysosomes, where they may interact with nucleic acids or target organelles [35]. Liu et al. in 1998 reported PSMA itself internalizes efficiently through clathrin-coated vesicles [36], and in metastatic prostate cancer cells, receptor expression is 100–1000 times higher than in normal prostate tissue [37]. Combining PSMA and TP in one molecule boosts cellular delivery, showing promise for advanced prostate cancer theranostics. Further mechanistic studies are needed to clarify each internalization pathway’s relative contribution and their impact on intracellular trafficking and nuclear targeting. 

[^64^Cu]Cu-NOTA-TP-PSMA showed greater intracellular retention in LNCap cells—most pronounced at 4 h—compared to [^64^Cu]Cu-NOTA-PSMA and [^64^Cu]Cu-NOTA-TP, likely reflecting the combined influence of both PSMA and TP ligands (Figure 4A). Similar results were observed with a PSMA ligand conjugate to a mitochondria-targeting triphenylphosphonium (TPP) moiety [38]. The combined hydrophobicity and positive charge of TPP enhanced intracellular and mitochondria-specific retention are key factors for more effective imaging and therapy. The reduced ligand efflux and improved trapping, also contribute to increase the effectiveness of [^64^Cu]Cu-NOTA-TP-PSMA **1**.

The significantly higher nuclear uptake of [^64^Cu]Cu-NOTA-TP-PSMA **1** compared to its monomeric counterparts reflects the anticipated synergy of the TP-PSMA conjugate (Figure 5, Table S4). By combining the DNA-intercalating properties of the TP moiety with the localized emission of low-energy electrons from ^64^Cu, [^64^Cu]Cu-NOTA-TP-PSMA **1** achieves enhanced nuclear delivery and retention in PSMA-positive LNCaP cells than ^64^Cu-NOTA-TP **2** or ^64^Cu-NOTA-PSMA **3**, reaching ~7%CPM/10^6^ cells at 24 h and maintaining elevated levels at 48 h. Minimal uptake in HEK-293 cells confirms PSMA-mediated specificity. Moreover, [^64^Cu]Cu-NOTA-TP-PSMA exhibited potent and low nanomolar cytotoxicity (EC_50_ = 10 nM) in PSMA-positive LNCaP cells, outperforming monomeric analogs (Table 3). Its selectivity index of ~25 at 24 h and ~10 at 48 h versus non-malignant HEK293 cells is predictive of precise tumor targeting. Importantly, cytotoxicity and nuclear localization are aligned, peaking at 24 h when [^64^Cu]Cu-NOTA-TP-PSMA **1** was ~5- and ~16-fold more potent than monomers **2** and **3**, mirroring ~13- and ~16-fold higher nuclear uptake. Cytotoxicity of **1** remained elevated at 48 h with sustained nuclear uptake, but both declined at 72 h with reduced retention. This correlation supports nuclear delivery as the driver of early cytotoxicity. 

Previous studies using STED nanoscopic, revealed that PSMA localized in the cytoplasm and, to a lesser extent, in perinuclear regions [39]. This predominant cytoplasmic distribution has been identified as a limitation of current PSMA ligands, such as ^177^Lu-PSMA-617, by restricting their ability to induce direct DNA damage [40,41]. In this regard, the improved nuclear delivery of [^64^Cu]Cu-NOTA-TP-PSMA **1**, specially as ^64^Cu as Auger emitter could explain its therapeutic effect when decay occurs near DNA [42]. The nanometer-to-micrometer range of these electrons necessitates precise nuclear localization to maximize efficacy while minimizing off-target toxicity [43], producing clustered double-strand breaks that overwhelm repair [44]. The TP motif likely amplifies this effect by binding G4 structures enriched in telomeres and promoters, thereby promoting nuclear trafficking [45] and directing the ^64^Cu payload to genomic DNA. 

Although ^64^Cu showed radiotherapeutic potential, it is not a pure Auger emitter and may therefore not represent the optimal radionuclide for therapeutic applications. Since we have demonstrated that our new radiotheranostic agent, [^64^Cu]Cu-NOTA-TP-PSMA **1**, is efficiently internalized and accumulates robustly within the nucleus of cancer cells, we believe that to further improve therapeutic efficacy, a pure Auger-electron emitter such as ^165^Er [46], ^119^Sb [47], or ^197^Hg [47] would likely be more appropriate. Additional studies will be required to identify suitable chelators capable of forming stable complexes with these radionuclides. In this context, the ^64^Cu/^165^Er, ^64^Cu/^119^Sb and ^64^Cu/^197^Hg theranostic pairs could offer translational advantages, enabling precise PET imaging with ^64^Cu and potent Auger-electron-based therapy using ^165^Er, ^119^Sb, or ^197^Hg.

Beyond direct DNA damage, TP complexes can induce mitochondrial dysfunction, ROS generation, and caspase-dependent apoptosis, offering a therapeutic edge in overcoming resistance to platinum drugs like cisplatin, which are limited by efflux and DNA repair [48]. Their twisted octahedral geometry further promotes DNA intercalation [49]. Importantly, ^64^Cu-NOTA-PSMA **3** lacking TP showed modest cytotoxicity despite efficient PSMA uptake, highlighting TP nuclear-active motif as a critical determinant to maximize radiocytotoxicity with Auger electrons [50].

The enhanced cytotoxicity of [^64^Cu]Cu-NOTA-TP-PSMA likely reflects a combination of mechanisms: TP-induced G4 destabilization synergizing with ^64^Cu-mediated DNA damage through Auger electrons, with nuclear localization supporting DNA damage as a primary driver. This combination not only produces clustered DNA lesions but is also expected to trigger downstream apoptosis, while oxidative stress may further contribute to cell death [51]. Mechanistic studies to delineate these pathways are currently underway and will be presented in a separate publication.

Overall, our results identify [^64^Cu]Cu-NOTA-TP-PSMA **1** as a promising radiotheranostic agent, warranting comprehensive in vivo evaluation to advance its development for PCa imaging and targeted radiotherapeutic applications

## 4. Materials and Methods

Unless otherwise specified, all reagents and solvents were used as provided by commercial suppliers without additional purification. The 2-chlorotrityl chloride resin was sourced from Chem-Impex International Inc. (Wood Dale, IL, USA). Fluorenylmethyloxycarbonyl (Fmoc)-protected amino acids were obtained from either EMD NovaBiochem (Gibbstown, NJ, USA) or Chem-Impex International Inc. 2,2′,2″-(1,4,7-triazacyclononane-1,4,7-triyl) triacetic acid (NOTA) derivative was purchased from CheMatech (Dijon, France). Organic solvents including acetonitrile (CH_3_CN), dichloromethane (DCM), *N*,*N*-dimethylformamide (DMF), and methanol (MeOH) were obtained from Fisher Scientific (Ottawa, ON, Canada). Prior to use, DMF was dried over 4 Å molecular sieves for a minimum of one week and filtered to remove residual amine contaminants.

All analytical instruments were routinely calibrated and maintained in accordance with internal quality assurance protocols. Mass spectrometry data were collected using multiple platforms: an API 3000 LC/MS/MS system (Applied Biosystems/MDS SCIEX, Concord, ON, Canada), a Waters/Alliance HT 2795 system equipped with a Waters 2996 photodiode array detector and a Waters Micromass ZQ mass spectrometer, an API 2000 instrument, and an ESI-Q-Tof (MAXIS).

NOTA-PSMA derivatives were purified using a Biotage HPFC SP4 Flash Purification System with a C18 column. Analytical high-performance liquid chromatography (HPLC) was carried out using an Agilent 1200 system (Agilent Technologies, Mississauga, ON, Canada) fitted with a Zorbax Eclipse XDB C18 reversed-phase column (4.6 × 250 mm, 5 μm) and a diode array UV-Vis detector. The elution protocol employed a linear gradient from 0 to 76.6% CH_3_CN in water (both solvents containing 0.05% trifluoroacetic acid) over 23 min, followed by a 1-min wash at 100% CH_3_CN, and a re-equilibration step returning to 0% CH_3_CN by 30 min. The flow rate was maintained at 1 mL/min.

Copper-64 (^64^Cu) was produced via the ^64^Ni(p,n)^64^Cu nuclear reaction using a TR-19 or TR-24 cyclotron (Advanced Cyclotron Systems Inc., ACSI, BC, Canada). The target material, ^64^Ni, was electroplated onto a rhodium backing. Following irradiation, ^64^Cu was isolated in the chloride form (^64^CuCl_2_) using a protocol adapted from McCarthy et al. and subsequently converted to the acetate form (^64^Cu(OAc)_2_) by dissolving the product in 0.1 M ammonium acetate buffer at pH 5.5 [52].

### 4.1. Synthesis, Radiolabeling, and Characterization 

The synthesis of the glutamate-urea-lysine motif and linker for PSMA peptides **4** and **5** was performed via standard solid-phase peptide synthesis (SPPS) as described by Benešová et al., with minor modifications (Scheme 1) [32]. Commercially available H-Lys(Alloc)-OH 2-chlorotrityl chloride resin (loading: 0.77 mmol/g) was pre-swollen in DCM for 30 min. Activation of the resin was achieved by adding *N*,*N*′-disuccinimidyl carbonate (DSC, 4 equiv) and DIPEA (8 equiv) in 20 mL DMF, stirring for 5 h at room temperature. After removal of the solution, the resin was washed thoroughly with DMF (3 × 20 mL). Coupling of H-Glu(OtBu)-HCl (2.5 equiv) and DIPEA (5 equiv) in DMF was carried out for 3 h at room temperature. Post-coupling, the resin was washed again with DMF (3 × 20 mL). Selective deprotection of the Alloc protecting group on the lysine side chain was performed using Pd(PPh_3_)_4_ (0.25 equiv) and morpholine (30 equiv) in DCM for 3 h. Residual palladium was removed by sequential washes: DIPEA–DMF (5:95, *v*/*v*) followed by sodium diethyldithiocarbamate in DMF. Coupling of Fmoc-D-2-Nal-OH was conducted using HATU (3.0 equiv) and DIPEA (6.0 equiv) in DMF for 3 h. Fmoc deprotection was performed using 20% piperidine in DMF (3 cycles of 10 min). Fmoc-tranexamic acid was coupled under identical conditions. Coupling efficiency at each step was monitored using the Kaiser test. Between each synthetic step, the resin was washed with DMF, DCM, and MeOH. Fmoc–Gly–OH (for peptide 2) and Fmoc–Lys(ivDde)–OH (for peptide 3) were coupled to the ε-amino group of lysine using HATU (5 equiv) and DIPEA (10 equiv) in DMF for 3 h. Subsequent Fmoc deprotection was carried out using 20% piperidine in DMF (2 × 10 min). Fmoc–PEG–OH was then coupled under the same conditions (HATU/DIPEA in DMF), followed by a final Fmoc removal step. After selective orthogonal deprotection, the NOTA-peptide conjugate was synthesized via bromoacetylation, introduction of triazacyclononane (TACN), and alkylation with tert-butyl bromoacetate, following previously reported protocols [53].

**NOTA-PSMA 4**: The resin was cleaved with a cocktail of TFA:H_2_O:triisopropylsilane (TIPS) (95:2.5:2.5) for 3 h. The resin was removed by filtration and washed with TFA. Combined filtrates were added dropwise to cold diethyl ether. The precipitated crude peptide was centrifuged, and the ether solution was decanted. Crude peptide **2** was purified by flash chromatography on a Biotage SP4 system, using a C18 cartridge. The product fractions were pooled and lyophilized to obtain the desired compound with 38% yield. Purity of the peptide was verified by HPLC and accounted for 99%; the identity was confirmed by API 3000 LC/MS/MS. HPLC: Retention time (Rt) = 14.80 min; ESI-MS: calcd: 1143.5, found, 1144.6 [M+1]^+^, 572.6 [M/2], (Figure S1).

**NOTA-TP-PSMA 5.** The ivDde protecting group on the lysine side chain of the resin-bound peptide was selectively removed by treatment with 2% hydrazine monohydrate in DMF for 30 min, followed by thorough washing with DMF. Subsequently, 2,2′:6′,2′′-terpyridine-4′-carboxylic acid [25] was coupled to the deprotected amine on-resin using HATU and DIPEA in DMF for 3 h. Final washes were carried out with DMF, DCM, and MeOH. Selective cleavage of the peptide from the resin was achieved using a mixture of trifluoroethanol (TFE) and DCM (1:2) for 3 h, affording a fully protected, coupling-ready PSMA derivative. Platination of the protected peptide was performed by treatment with 2 equivalents of K_2_PtCl_4_ in methanol at 60 °C under gentle agitation for 2 h. After evaporation of methanol, the crude product was treated with neat TFA for 2 h and subsequently purified by Biotage flash chromatography to yield NOTA-TP-PSMA **5** in 36% yield. It is important to note that the purification should be carried out promptly, and the acidic solution should not be left to stand for too long to prevent the decomposition of the platinum–peptide. Peptide purity was confirmed by analytical reverse-phase HPLC (Rt = 14.99 min), and identity was verified by ESI-MS (calcd: 1702.6; found: 1703.8 [M+H]^+^, 852.5 [M/2] (Figure S2). 

For the synthesis of ^nat^Cu-NOTA-PSMA and ^nat^Cu-NOTA-TP-PSMA, peptides **4** and **5** were first solubilized in a minimal amount of DMSO. Complexation was initiated by the addition of 1.1 equivalents of high-purity Cu(OAc)_2_, prepared in 0.1 M ammonium acetate buffer at pH 5.5, allowing the formation of the copper-labeled conjugates under mild aqueous conditions.

**^nat^Cu-NOTA-PSMA 2** After stirring at room temperature for 30 min, the reaction mixture changed from white to green, indicating complex formation. The product was purified using a C18 Sep-Pak cartridge and eluted with ethanol, yielding the desired pale green complex with 88% yield and 99% purity. Analytical HPLC showed a retention time of 14.90 min, and ESI-MS confirmed the expected mass (calcd: 1205.3; found: 1206.3 [M+H]^+^, 602.5 [M/2] (Figure S3). 

**^nat^Cu-NOTA-TP-PSMA 3** Within one hour of stirring at room temperature, the reaction mixture evolved from orange to green, leading to the formation of a green precipitate. This solid was isolated by filtration, thoroughly rinsed with water and cold ether, and dried to yield the product as a green solid with 89% yield. Analytical HPLC showed a retention time of 14.56 min, ESI-MS: calcd 1763.2; found, 1764.0 [M+1], 882.5 [M/2], 588.7.0 [M/3],441.9 [M/4], Figure S4.

**Radiobeling** of NOTA-TP-PSMA, NOTA-PSMA, and NOTA-TP was achieved by incubating 5–10 nmol of each precursor with 400–1000 MBq of [^64^Cu]Cu(OAc)_2_ in 500 μL of 0.1 M ammonium acetate buffer (pH 7.2) at room temperature for 20 min. Radiochemical purity and labeling efficiency were evaluated by radio-instant thin-layer chromatography (radio-iTLC) using C18 silica gel plates and 0.1 M sodium citrate buffer (pH 5.5) as the mobile phase. Radioactive species were visualized using an Instant Imager system (BioScan, Washington, DC, USA). Under these conditions, free [^64^Cu]Cu^2+^ migrated with the solvent front, whereas the radiolabeled [^64^Cu]Cu–NOTA conjugates remained at the origin (Figure S5).

### 4.2. Stability Studies

The in vitro plasma stability of [^64^Cu]Cu-NOTA-TP-PSMA 1 was evaluated by incubating 250 MBq of the radiolabeled compound (in 250 μL PBS) with 250 μL of freshly collected mouse plasma at 37 °C for up to 24 h. At predefined time points, (1 h, 4 h and 24 h) plasma proteins were precipitated by adding ethanol (1:1, *v*/*v*) in two successive steps, followed by vortexing (1 min) and centrifugation at 7000 rpm for 10 min. The resulting supernatant was subjected to ultracentrifugation to ensure complete removal of particulate matter.

For assessment of in vitro stability, the clarified supernatant was analyzed by radio-instant thin-layer chromatography (radio-iTLC) on C18 silica gel plates, using 0.1 M sodium citrate buffer (pH 5.5) as the mobile phase. Radiodetection was performed using an Instant Imager system (BioScan, Washington, DC, USA). Free [^64^Cu]Cu(OAc)_2_ and intact [^64^Cu]Cu-NOTA-TP-PSMA served as controls to facilitate peak identification. Under these chromatographic conditions, the radiolabeled complex remained at the origin, while free copper migrated with the solvent front (Figure S5).

To quantify the extent of protein binding, radioactivity in both the protein pellet and the supernatant was measured using a dose calibrator. The percentage of protein-bound radioactivity was calculated by comparing the activity in the precipitated fraction relative to the total recovered activity. This analysis provided complementary information regarding the in vitro stability and biodistribution-relevant binding profile of the radiotracer.

### 4.3. Cell Culture

Lymph node carcinoma of the prostate (LNCaP) cells, which express prostate-specific membrane antigen (PSMA), and human embryonic kidney (HEK-293) cells, a PSMA-negative control, were obtained from the American Type Culture Collection (ATCC, Manassas, VA, USA). LNCaP cells were cultured in RPMI-1640 medium and HEK-293 cells in EMEM medium (Gibco, Waltham, MA, USA). Both media were supplemented with 10% fetal bovine serum (FBS; Sigma-Aldrich, Waltham, MA, USA), 1% penicillin-streptomycin (Gibco, USA), and 1% amphotericin B (Sigma-Aldrich, USA). Cells were maintained at 37 °C in a humidified 5% CO_2_ incubator.

### 4.4. Competition Affinity Binding Assays in LNCaP Cells

To determine binding affinity, 100,000 LNCaP cells were seeded in 12-well plates and incubated for 72 h to 80–90% confluency. Before the assay, the medium was replaced with RPMI-1640 containing 2% HEPES, 20 g/L bovine serum albumin (BSA), and 1% penicillin-streptomycin. Cells were incubated for 1 h at 37 °C with 7.5 nM [^64^Cu]Cu-NOTA-PSMA in the presence of increasing concentrations (10^−3^ to 10^−13^ M) of ^nat^Cu-NOTA-TP-PSMA and ^nat^Cu-NOTA-PSMA.

After incubation, cells were washed with phosphate-buffered saline (PBS; Gibco, USA), detached using trypsin (Gibco, USA), and radioactivity was measured using a gamma counter (HIDEX, Turku, Finland). All assays were performed in triplicate and all data presented in Results are the average of three independent experiments. IC_50_ values were calculated by nonlinear regression (log[inhibitor] vs. response—variable slope) using GraphPad Prism 10 (GraphPad Software, San Diego, CA, USA). Data are expressed as mean ± standard deviation (SD).

### 4.5. Cellular Uptake, Internalization, and Efflux Studies in LNCaP Cells

LNCaP cells (1 × 10^5^ cells/well) were seeded into 24-well plates and incubated for 72 h. Cells were treated with 1nM [^64^Cu]Cu-NOTA-TP-PSMA or control monomers ([^64^Cu]Cu-NOTA-TP and [^64^Cu]Cu-NOTA-PSMA), each containing 0.1 MBq of radioactivity in RPMI-1640 medium.

Uptake and internalization were assessed at multiple time points (30 min to 24 h). At each timepoint, cells were washed with cold PBS, detached with trypsin, and counted using a hemocytometer. For internalization studies, surface-bound radioactivity was removed using 50 mM glycine-HCl buffer (pH 2.2) for 5 min, followed by additional PBS washes.

To investigate the internalization mechanism, parallel experiments were conducted at 4 °C to evaluate passive versus active uptake. Receptor specificity was assessed via blocking assays using 1 µM ^nat^Cu-NOTA-TP-PSMA (1000-fold excess) co-incubated with 1 nM radiolabeled tracer. Post-incubation, intracellular radioactivity was quantified using a HIDEX gamma counter. All results were normalized to 10^6^ cells and expressed as percent counts per minute per million cells (%CPM/10^6^ cells).

Efflux studies were conducted by incubating the cells with the compound for an initial period of 1 h, after which the incubation medium was replaced with fresh, compound-free medium. The retained intracellular radioactivity was then measured at various time points up to 24 h and expressed as the percentage of retention relative to the 0-h time point, defined as the time immediately after medium replacement.

### 4.6. Subcellular Localization

To assess nuclear localization, LNCaP and HEK-293 cells were incubated with 1 MBq of [^64^Cu]Cu-NOTA-TP-PSMA **1** for 24, 48, and 72 h. Nuclear extraction was performed following a previously established protocol [25]. Following incubation, cells were lysed and fractionated using cytoskeletal buffer to isolate nuclear components. Nuclear and cytoplasmic radioactivity were measured using a HIDEX automatic gamma counter. Data were used to evaluate time-dependent nuclear accumulation of the tracer.

### 4.7. Cytotoxicity Assay in LNCaP and HEK-293 Cells 

Cytotoxicity was evaluated using a resazurin-based assay (PrestoBlue) in 96-well plates. LNCaP (15,000 cells/well) and HEK-293 (10,000 cells/well) were seeded and incubated for 72 h. Cells were then treated with serial dilutions of [^64^Cu]Cu-NOTA-TP-PSMA and controls for 24, 48, or 72 h. After treatment, cells were rinsed with PBS and incubated with PrestoBlue reagent for 40 min. Fluorescence was measured at 570 nm (excitation) and 610 nm (emission). Background readings from blank wells were subtracted. Cell viability was calculated relative to untreated controls. IC_50_ values were determined using dose–response curves. Controls included [^64^Cu]Cu-NOTA-TP, [^64^Cu]Cu-NOTA-PSMA, ^nat^Cu-NOTA-TP-PSMA, and cisplatin (0–500 µM in 0.9% saline). All assays were performed in triplicate and all data presented in Results are the average of three independent experiments. TP conjugates were dissolved in 1% DMSO and diluted in saline. Inhibitory concentration 50 (IC_50_) values were calculated to quantify the effectiveness of the compounds.

### 4.8. Data Analysis

All experiments were performed in triplicate and repeated independently at least three times. Results are expressed as mean ± SD. Statistical comparisons were conducted using Welch’s *t*-test, with *p* < 0.05 considered statistically significant. Data analysis was performed using GraphPad Prism 7.0 (GraphPad Software, La Jolla, CA, USA). 

## 5. Conclusions

In conclusion, [^64^Cu]Cu-NOTA-TP-PSMA demonstrates enhanced specificity, internalization, retention, subcellular localization, and cytotoxic potency over monomeric radioligands, highlighting it as a compelling candidate for advanced PSMA-targeted radiotheranostics. Future studies should aim to elucidate the radiobiological mechanisms underlying the enhance in vitro cytotoxicity of [^64^Cu]Cu-NOTA-TP-PSMA and to assess its potential as a targeted radioligand for prostate cancer imaging and therapy.

## Figures and Tables

**Table 1:** Inhibitory concentration (IC_50_) of PSMA ligands in LNCaP cells as determined by competitive binding assay. Data expressed as mean ± SD from three independent replicates.

Entry	PSMA Ligand	IC_50_ (nM)
entry 1	^nat^Cu-NOTA-PSMA	40.8 ± 14.9
entry 2	^nat^Cu-NOTA-TP-PSMA	30.6 ± 16.6

All values are presented as mean ± standard deviation from three independent experiments.

**Table 2:** Comparative uptake and internalization of ^64^Cu-labeled conjugates in LNCaP cells at 24 h. Values are expressed as %CPM/10^6^ cells.

Entry	Radioconjugate	Uptake	Internalization
1	[^64^Cu]Cu-NOTA-TP-PSMA **1**	65.9 ± 8.6	44.9 ± 7.3
2	[^64^Cu]Cu-NOTA-TP **2**	39.5 ± 8.0	36.1 ± 9.2
3	[^64^Cu]Cu-NOTA-PSMA **3**	22.8 ± 1.2	16.7 ± 1.6

**Table 3:** EC_50_ (nM) of compounds in LNCaP and HEK-293 cells at 24, 48, and 72 h.

Entry	Compound	24 h		48 h		72 h	
		LNCaP	HEK293	LNCaP	HEK293	LNCaP	HEK293
1	[^64^Cu]Cu-NOTA-TP-PSMA **1**	10 ± 2	>250	24 ± 6	>250	24 ± 8	N/A
2	[^64^Cu]Cu-NOTA-TP **2**	53 ± 4	N/A	47 ± 4	N/A	278 ± 5	N/A
3	[^64^Cu]Cu-NOTA-PSMA **3**	159 ± 7	204 ± 7	103 ± 16	195 ± 10	93 ± 14	149 ± 6
4	^nat^Cu-NOTA-TP-PSMA	18,504 ± 4	42,199 ± 8	17,780 ± 6	23,402 ± 8	16,693 ± 4	17,647 ± 4
5	Cisplatin	81,006 ± 14	31,257 ± 3	30,412 ± 6	41,023 ± 7	22,859 ± 5	27,481 ± 11

(mean ± SD; n = 3–5). N/A = not available.

## Data Availability

All data generated or analyzed during this study are either included in this published article and its Supplementary Information files or are available from the corresponding author upon reasonable request.

## Supplementary Materials

The following supporting information can be downloaded at: https://www.mdpi.com/article/10.3390/ijms262311651/s1.

## Abbreviations

The following abbreviations are used in this manuscript:

^225^Ac	Actinium-225
^68^Ga	Gallium-68
^77^Lu	Lutetium-177
LD	Linear dichroism
BSA	Bovine serum albumin
CH_3_CN	Acetonitrile
CPM	Counts per minute
Cu(OAc)_2_	Copper(II) acetate
CuCl_2_	Copper(II) chloride
DCM	Dichloromethane
DIPEA	*N*,*N*-diisopropylethylamine
DMSO	Dimethyl sulfoxide
DNA	Deoxyribonucleic acid
DSC	*N*,*N*′-Disuccinimidyl carbonate
EC_50_	Half-maximal effective concentration
EDC	1-(3-Dimethylaminopropyl)-3-ethylcarbodiimide hydrochloride
EMEM	Eagle’s minimum essential medium
ESI-Q-Tof	Electrospray ionization quadrupole time-of-flight
FBS	Fetal bovine serum
FDA	Food and Drug Administration
Fmoc	Fluorenylmethyloxycarbonyl
G4	G-quadruplex
HATU	*O*-(7-azabenzotriazol-1-yl)-N,N,N′,N′-tetramethyluronium hexafluorophosphate
HEK-293	Human embryonic kidney 293 cells
HEPES	4-(2-Hydroxyethyl)-1-piperazineethanesulfonic acid
HPLC	High-performance liquid chromatography
IC_50_	Half-maximal inhibitory concentration
iTLC	Instant thin-layer chromatography
ivDde	1-(4,4-Dimethyl-2,6-dioxocyclohexylidene)ethyl
LC/MS/MS	Liquid chromatography tandem mass spectrometry
LEEs	Low-energy electrons
LET	Linear energy transfer
LNCaP	Lymph Node Carcinoma of the Prostate
MBq	Megabecquerel
MBq/nmol	Megabecquerel per nanomole
mCRPC	Metastatic castration-resistant prostate cancer
MeOH	Methanol
NOTA	2,2′,2″-(1,4,7-triazacyclononane-1,4,7-triyl)triacetic acid
PBS	Phosphate-buffered saline
PCa	Prostate cancer
PEG	Polyethylene glycol
PET	Positron emission tomography
PSA	Prostate-specific antigen
PSMA	Prostate-specific membrane antigen
RLT	Radioligand therapy
RNA	Ribonucleic acid
ROS	Reactive oxygen species
RPMI-1640	Roswell Park Memorial Institute 1640 medium
Rt	Retention time
SD	standard deviation
SPPS	Solid-phase peptide synthesis
TACN	Triazacyclononane
TFA	Trifluoroacetic acid
TFE	Trifluoroethanol
TIPS	Triisopropylsilane
TP	Terpyridine–platinum
