# Supplementary material for: Development and In Vitro Evaluation of [64Cu]Cu-NOTA-TP-PSMA, a Novel Radiotheranostic Agent Against Prostate Cancer
Source: Int J Mol Sci. 2025 Dec 1;26(23):11651. doi: 10.3390/ijms262311651 (PMC12692291; doi:10.3390/ijms262311651)
Supplement: Supplementary file 1 [file ijms-26-11651-s001.zip › ijms-3983823-supplementary.pdf]

SUPPLEMENTAL INFORMATION

Development and In Vitro Evaluation of [^{64}Cu]Cu-NOTA-TP-PSMA, a Novel Radiotheranostic Agent Against Prostate Cancer

Hoda Talebian, Samia Ait-Mohand, Prenitha Mercy Ignatius Arokia Doss, Léon Sanche, and Brigitte Guérin*

Supplemantary data

Table of content

**Figure S1.** ESI-MS spectrum spectrum and HPLC chromatogram of NOTA-PSMA **4**	S2
**Figure S2.** ESI-MS spectrum and HPLC chromatogram of NOTA-TP-PSMA **5**	S3
**Figure S3**. ESI-MS spectrum and HPLC chromatogram of ^{nat}Cu-NOTA-PSMA	S4
**Figure S4.** ESI-MS spectrum and HPLC chromatogram of ^{nat}Cu-NOTA-TP-PSMA	S5
**Figure S5.** Radio-TLC of the radiolabelling and the plasma stability of [^{64}Cu]Cu-NOTA-TP-PSMA. A) ^{64}Cu (free); B) [^{64}Cu]Cu-NOTA-TP-PSMA; C) [^{64}Cu]Cu-NOTA-TP-PSMA after 24 h incubation in plasma.	S6
**Figure S6.** Inhibition of [^{64}Cu]Cu-NOTA-PSMA binding to PSMA on LNCap cells with various concentrations of ^{nat}Cu-NOTA-PSMA and ^{nat}Cu-NOTA-TP-PSMA	S6
**Figure S7.** Cytotoxicity of ^{64}Cu-NOTA conjugates on LNCap and HECK293 cells cells assessed by PrestoBlue assay at 24 (red line), 48 (green line) and 72 h (black line).	S7
**Table S1.** Statistical comparison of time-dependent uptake vs. blocking conditions of ^{64}Cu-NOTA-TP-PSMA (unpaired *t*-tests). Data are expressed as mean ± SD (%ID/10^6 cells).	S8
**Table S2.** Time-dependent uptake and internalization of ^{64}Cu-NOTA-TP-PSMA **1** in PSMA-positive (LNCaP) and PSMA-negative (HEK-293) cells. Data are expressed as mean ± SD (%ID/10^6 cells).	S8
**Table S3.** Retained activity (%CPM/10^6 cells) of ^{64}Cu-labeled conjugates over 24 hours in LNCaP vs HEK-293 cells.	S8
**Table S4.** Nuclear uptake values of radioconjugate **1**, **2** and **3** in LNCaP and HEK-293 at 24-, 48-, and 72-hours post incubation and statistical comparison(unpaired *t*-tests). Data are expressed as mean ± SD (%ID/10^6 cells).	S9

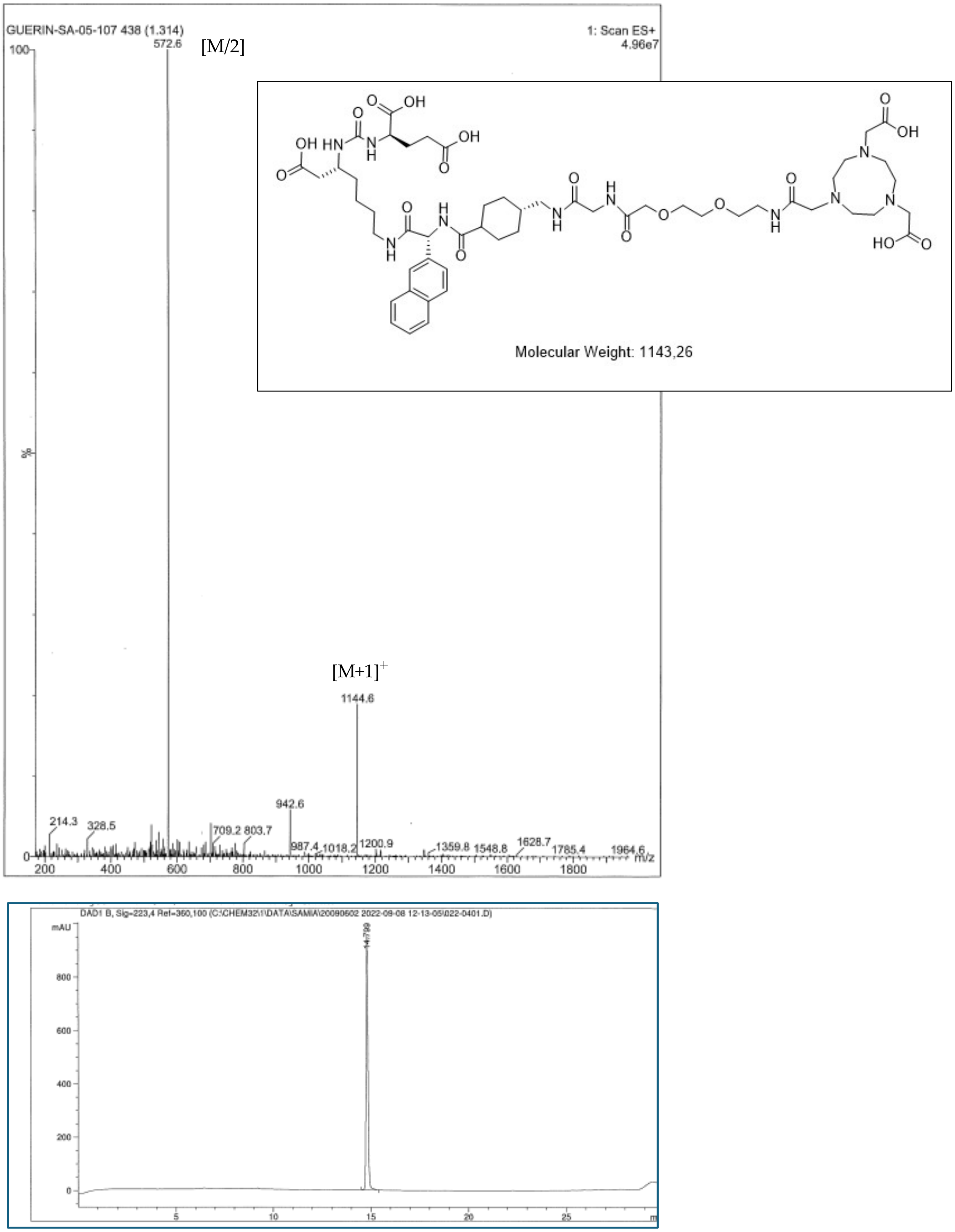

Figure S1. ESI-MS spectrum and HPLC chromatogram of NOTA-PSMA **4**

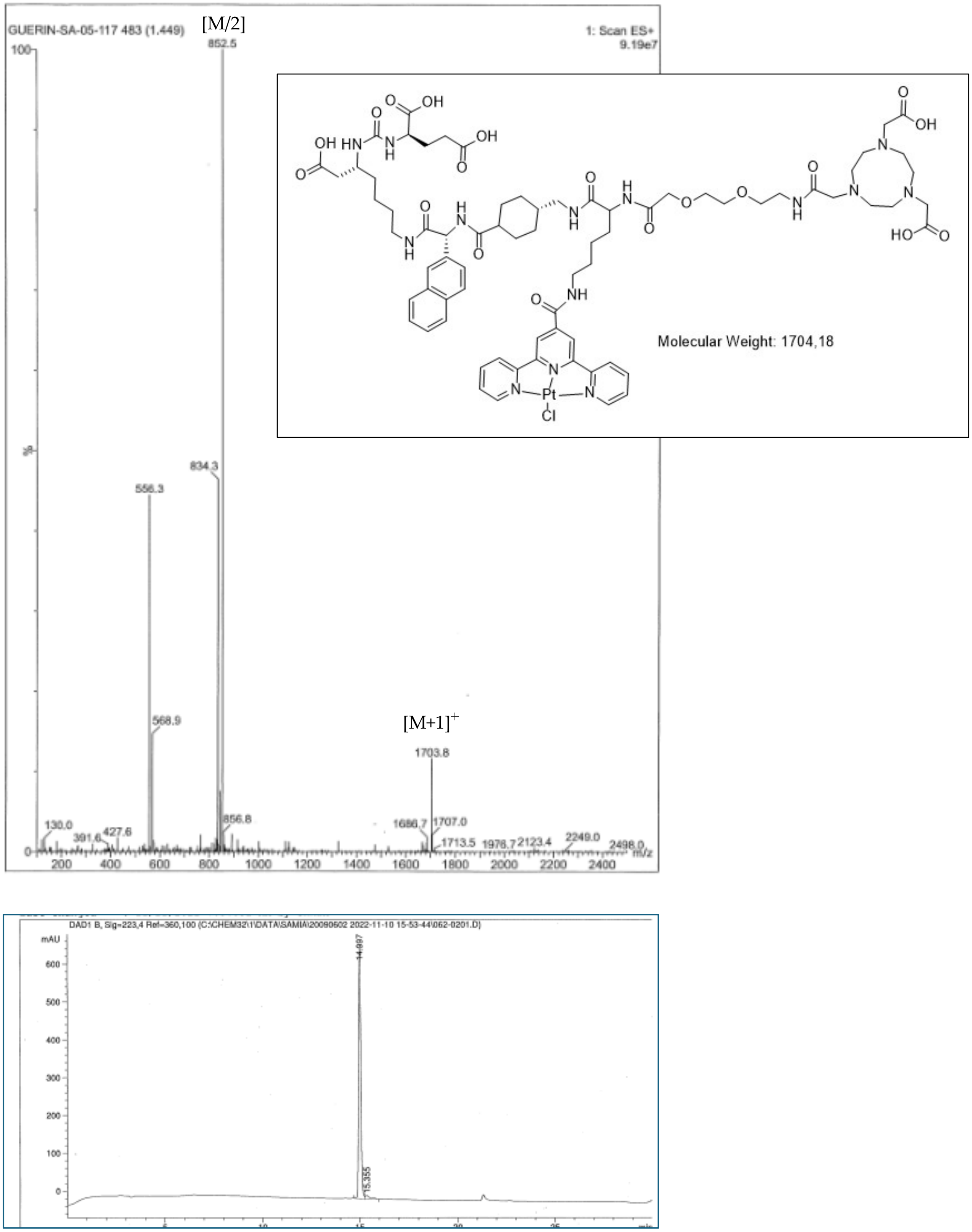

Figure S2. ESI-MS spectrum and HPLC chromatogram of NOTA-TP-PSMA **5**

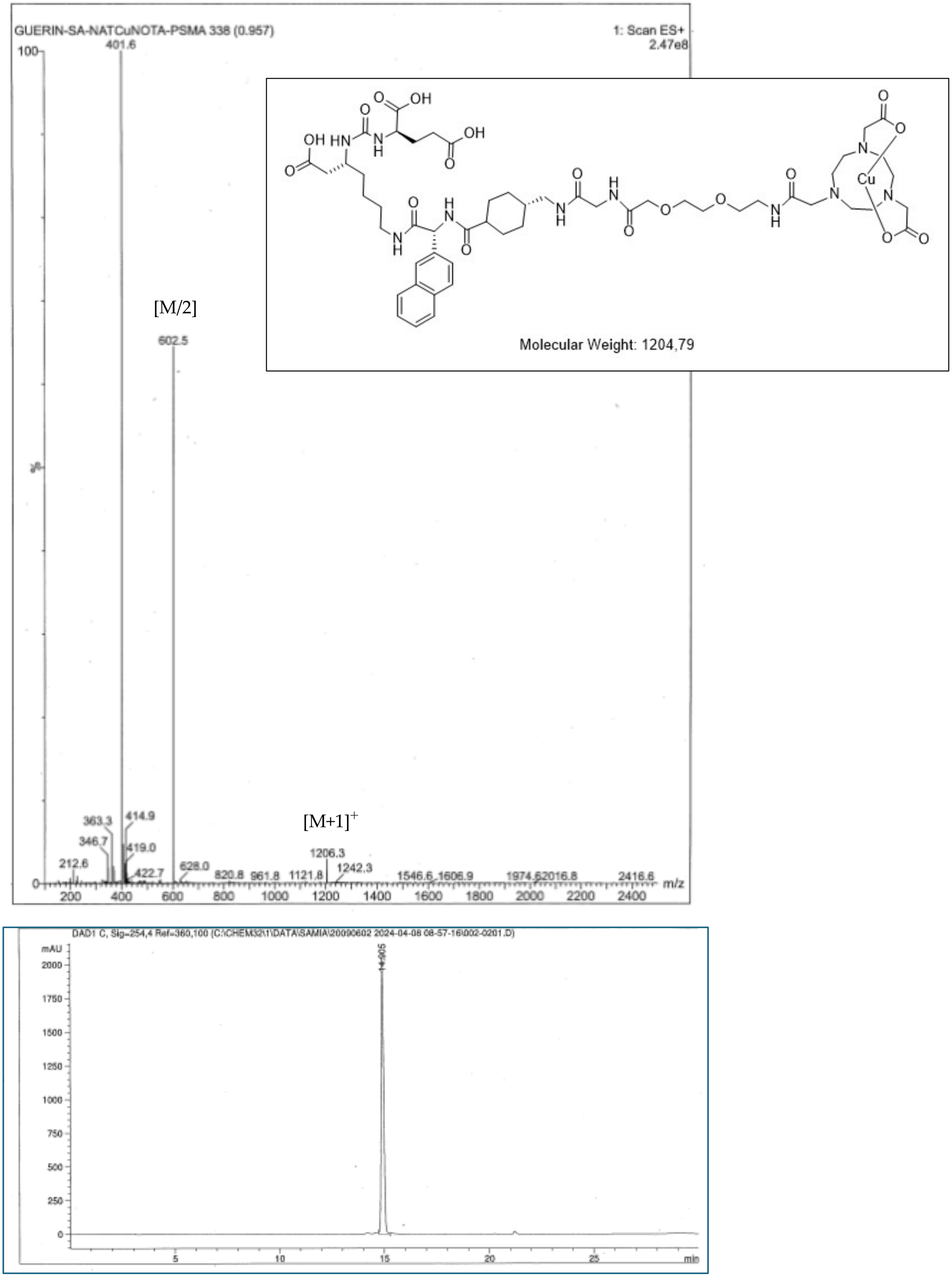

Figure S3. ESI-MS spectrum and HPLC chromatogram of ^{nat}Cu-NOTA-PSMA

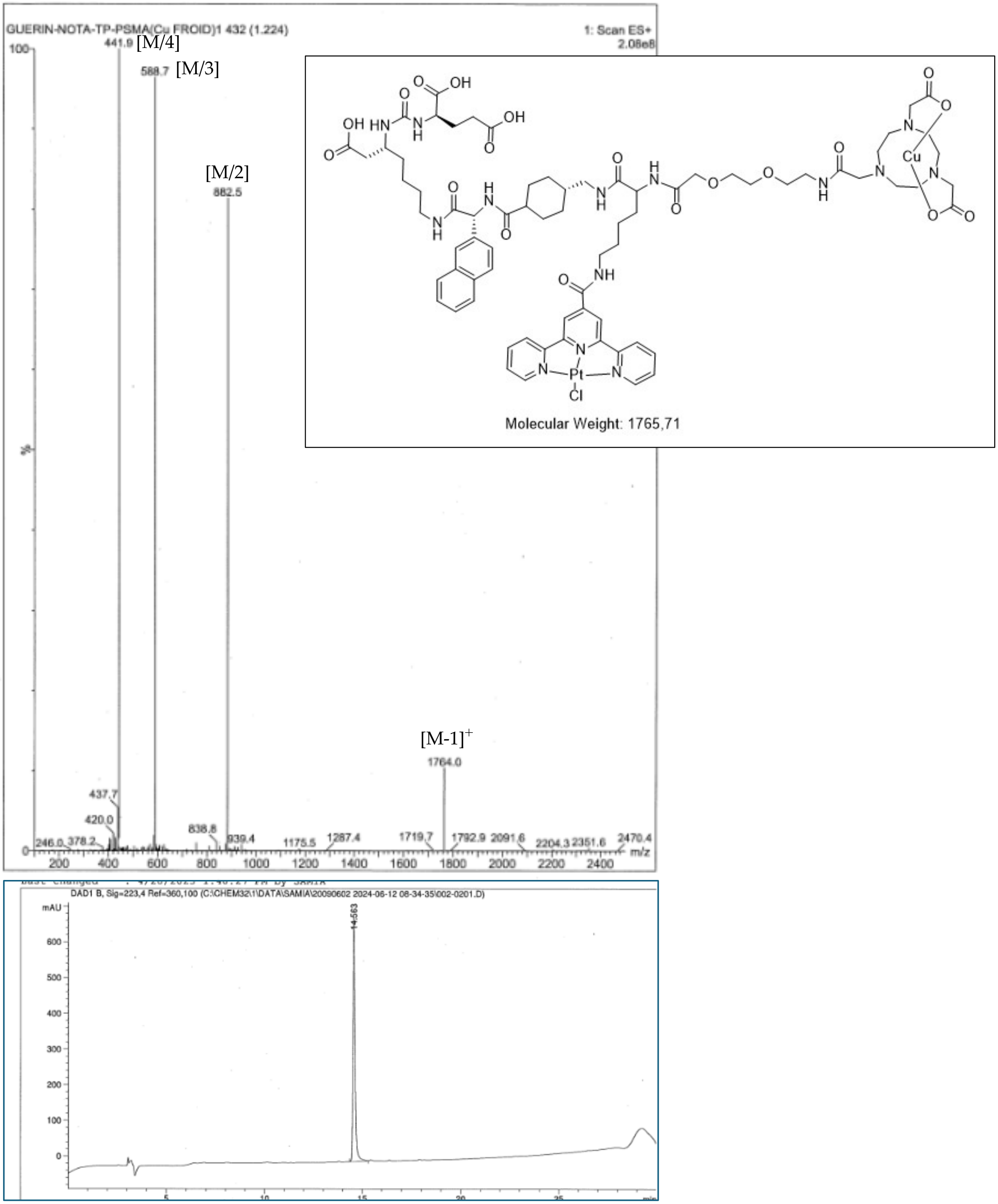

Figure S4. ESI-MS spectrum and HPLC chromatogram of ^{nat}Cu-NOTA-TP-PSMA

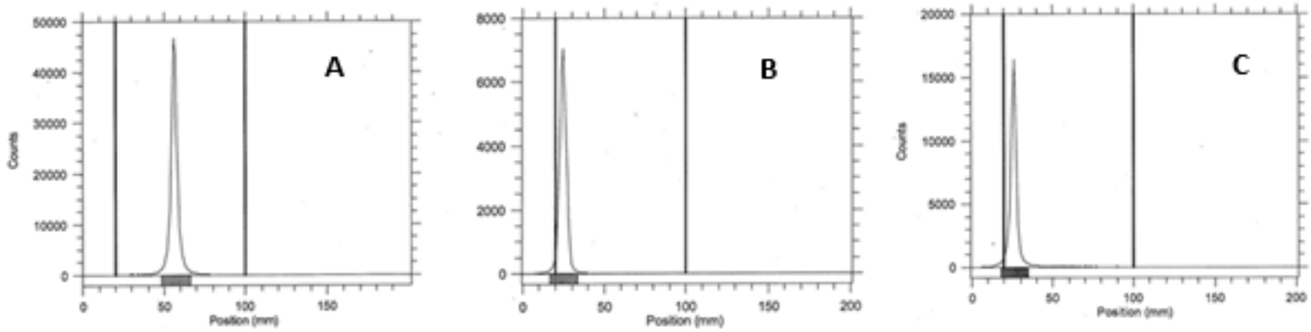

Figure S5. Radio-TLC of the radiolabelling and the plasma stability of [^{64}Cu]Cu-NOTA-TP-PSMA. A) ^{64}Cu (free); B) [^{64}Cu]Cu-NOTA-TP-PSMA; C) [^{64}Cu]Cu-NOTA-TP-PSMA after 24 h incubation in plasma.

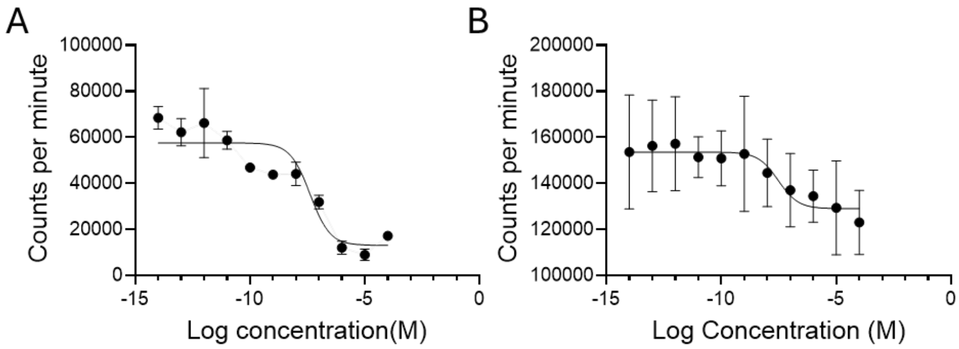

Figure S6. Inhibition of [^{64}Cu]Cu-NOTA-PSMA binding to PSMA on LNCap cells with various concentrations of ^{nat}Cu-NOTA-PSMA (A) and ^{nat}Cu-NOTA-TP-PSMA (B)

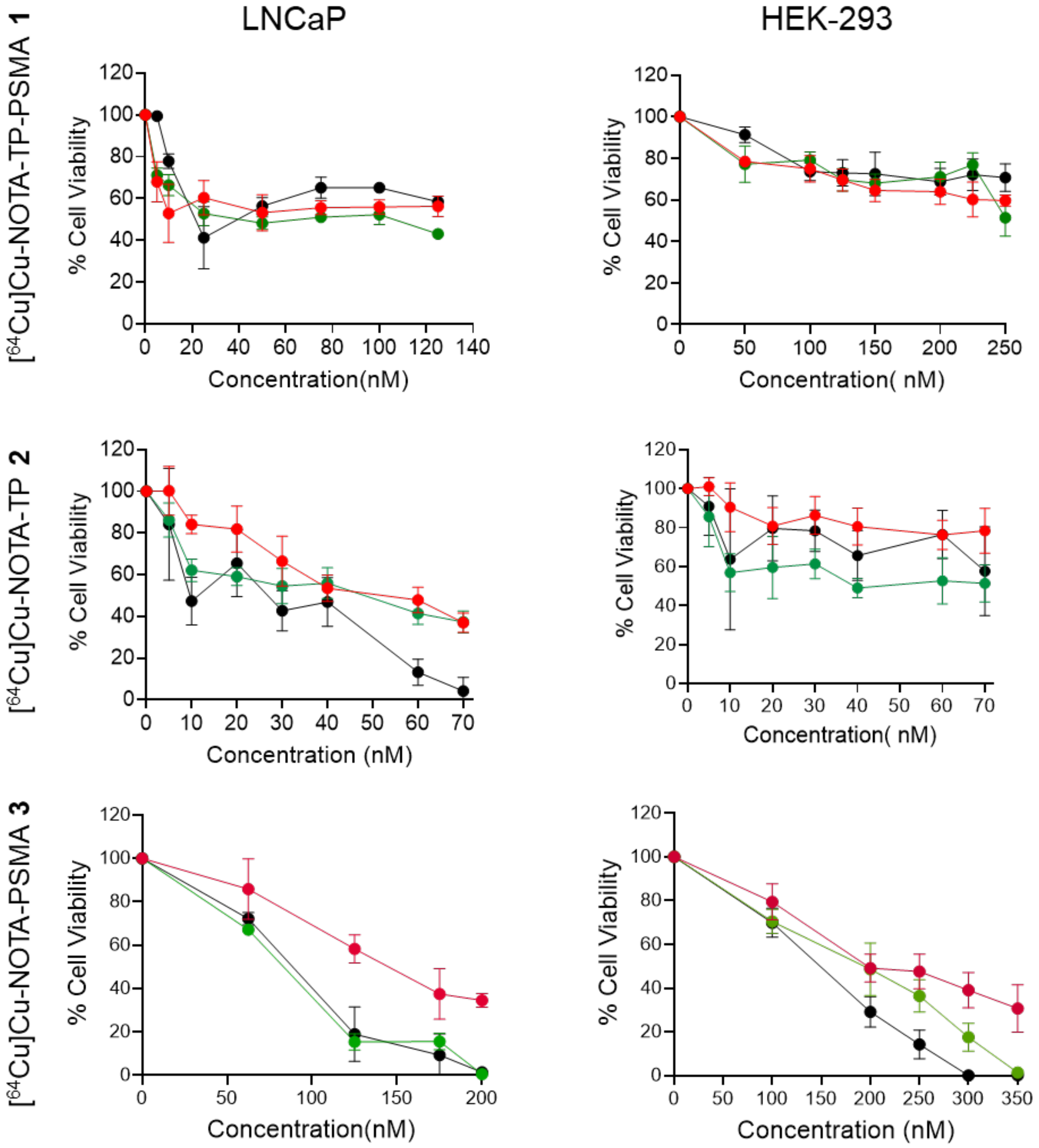

Figure S7. Cytotoxicity of ^{64}Cu-NOTA conjugates on LNCap and HECK293 cells cells assessed by PrestoBlue assay at 24 (red line), 48 (green line) and 72 h (black line).

Table S1. Statistical comparison of time-dependent uptake vs. blocking conditions of ^{64}Cu-NOTA-TP-PSMA (unpaired *t*-tests Data are expressed as mean ± SD (%ID/10^6 cells).

Time (h)	Uptake	Uptake blockage	p Value
1	16.52 ± 5.38	11.06 ± 5.93	p = 0.0073
2	30.57 ± 6.09	16.36 ± 7.23	p = 0.000001
4	44.61 ± 16.49	18.70 ± 10.46	p = 0.00001
20	62.88 ± 9.10	30.68 ± 11.43	p < 0.000001
24	65.87 ± 8.37	37.00 ± 13.09	p < 0.000001

n = 16 -18; *: p < 0.05, **: p < 0.01, ***: p < 0.001, ****:p < 0.0001

Table S2. Time-dependent uptake and internalization of ^{64}Cu-NOTA-TP-PSMA **1** in PSMA-positive (LNCaP) and PSMA-negative (HEK-293) cells. Data are expressed as mean ± SD (%ID/10^6 cells).

Time (h)	Uptake			Internalization		
	LNCaP	HEK-293	p-value*	LNCaP	HEK-293	p-value*
1	16.52 ± 5.53	3.15 ± 0.53	p < 0.000001	11.91 ± 5.75	4.46 ± 6.12	p = 0.0028
2	28.67 ± 9.74	3.78 ± 0.95	p < 0.000001	17.07 ± 6.19	3.34 ± 1.41	p < 0.000001
4	44.61 ± 16.99	5.27 ± 1.00	p < 0.000001	30.27 ± 8.92	3.26 ± 0.44	p < 0.000001
20	62.88 ± 9.37	8.00 ± 2.25	p < 0.000001	42.49 ± 9.41	6.12 ± 0.88	p < 0.000001
24	65.87 ± 8.61	8.30 ± 1.63	p < 0.000001	44.94 ± 7.32	7.65 ± 1.67	p < 0.000001

n = 8 -18, * LNCaP vs HEK-293; *: p < 0.05, **: p < 0.01, ***: p < 0.001, ****:p < 0.0001

Table S3. Retained activity (%CPM/10^6 cells) of ^{64}Cu-labeled conjugates over 24 hours in LNCaP vs HEK-293 cells.

Time (h)	^{64}Cu-NOTA-TP-PSMA	^{64}Cu-NOTA-TP	^{64}Cu-NOTA-PSMA
0	100 ± 0	100 ± 0	100 ± 0
1	85.36 ± 11.10	50.08 ± 9.30	54.8 ± 10.0
2	71.36 ± 13.48	42.9 ± 7.9	49.6 ± 8.1
4	67.8 ± 9.3	37.2 ± 8.9	46.6 ± 8.1
24	51.7 ± 9.4	38.8 ± 6.5	39.2 ± 7.9
0	100 ± 0	100 ± 0	100 ± 0
1	97.8 ± 4.3	19.2 ± 4.3	59.4 ± 22.3
2	62.6 ± 18.6	13.2 ± 15.9	45.1 ± 14.5
4	41.5 ± 9.9	10.0 ± 8.3	38.4 ± 4.1
24	17.1 ± 1.9	6.9 ± 0.8	22.0 ± 1.5

n= 6-12

Table S4. Nuclear uptake values of radioconjugate **1**, **2** and **3** in LNCaP and HEK-293 at 24-, 48-, and 72-hours post incubation and statistical comparison. Data are expressed as mean ± SD (%ID/10^6 cells).

Time (h)	^{64}Cu-NOTA-TP-PSMA 1		p-value LNCaP vs HEK-293	^{64}Cu-NOTA-TP 2		p-value LNCaP vs HEK-293	^{64}Cu-NOTA-PSMA 3		p-value‡ LNCaP vs HEK-293	p-value 1 vs 2 in LNCap	p-value 1 vs 3 in LNCap
	LNCaP	HEK-293		LNCaP	HEK-293		LNCaP	HEK-293			
24	6.96 ± 1.97	0.08 ± 0.05	p = 0.000023	0.54 ± 0.26	0.26 ± 0.08	p = 0.027	0.44 ± 0.25	0.07 ± 0.05	p = 0.0026	p = 0.000031	p = 0.000029
48	5.66 ± 2.09	0.15 ± 0.06	p = 0.000047	1.08 ± 0.35	0.33 ± 0.07	p = 0.00017	0.77 ± 1.02	0.13 ± 0.05	p = 0.096	p = 0.00015	p = 0.000045
72	1.53 ± 0.38	0.85 ± 0.84	p = 0.048	1.05 ± 0.34	0.40 ± 0.09	p = 0.000371	0.35 ± 0.07	0.23 ± 0.04	p = 0.000482	p = 0.013	p = 0.000012

*: $p < 0.05$, **: $p < 0.01$, ***: $p < 0.001$, ****: $p < 0.0001$
